# Nasal Airflow Dynamics following LeFort I Advancement in Cleft Nasal Deformities: A Retrospective Preliminary Study

**DOI:** 10.3390/diagnostics14121294

**Published:** 2024-06-19

**Authors:** Daniel Charles, Lucas Harrison, Fatemeh Hassanipour, Rami R. Hallac

**Affiliations:** 1 Department of Mechanical Engineering, University of Texas at Dallas, Richardson, TX 75080, USA; fatemeh@utdallas.edu; 2 Department of Plastic and Craniofacial Surgery, UT Southwestern Medical Center, Dallas, TX 75390, USA; lucas.harrison@utsouthwestern.edu (L.H.); rami.hallac@childrens.com (R.R.H.); 3 Analytical Imaging and Modeling Center, Children’s Medical Center Dallas, Dallas, TX 75235, USA

**Keywords:** cleft lip, LeFort I advancement, computational fluid dynamics

## Abstract

Unilateral cleft lip and palate (UCLP) nasal deformity impacts airflow patterns and pressure distribution, leading to nasal breathing difficulties. This study aims to create an integrated approach using computer-aided design (CAD) and computational fluid dynamics (CFD) to simulate airway function and assess outcomes in nasal deformities associated with unilateral cleft lip and palate (UCLP) after LeFort I osteotomy advancement. Significant alterations were observed in nasal geometry, airflow velocity, pressure dynamics, volumetric flow rate, and nasal resistance postoperatively, indicating improved nasal airflow. The cross-sectional area increased by 26.6%, airflow rate by 6.53%, and nasal resistance decreased by 6.23%. The study offers quantitative insights into the functional impacts of such surgical interventions, contributing to a deeper understanding of UCLP nasal deformity treatment and providing objective metrics for assessing surgical outcome.

## 1. Introduction

Unilateral cleft lip and palate (UCLP) represents a significant congenital deformity that affects both the structure and function of the oral and nasal cavities. Cleft lip and palate, even in mild forms, can cause various degrees of nasal deformities [1]. This condition often leads to a series of complications, including difficulties in speech and feeding, impaired nasal breathing, and impaired maxillary growth [2,3,4]. These difficulties may be related to smaller cross-sectional areas and reduced nasal airway volumes in patients with UCLP. Acoustic rhinometry studies demonstrated significantly smaller cross-sectional areas on the cleft side, confirming the persistence of nasal deformities and impaired patency even post-repair in UCLP children [5].

Addressing these challenges often requires surgical interventions, with many patients undergoing LeFort I osteotomy for maxillary advancement prior to secondary rhinoplasty [6,7]. Between 25% and 60% of patients with cleft lip and palate require some form of maxillary advancement surgery for effective midface correction [8]. The procedure involves a horizontal osteotomy across the maxilla, extending through the nasal floor and the base of the maxillary sinuses [9]. When the maxilla is mobilized, it is advanced to an optimal position, altering the anatomy of the nasal airways in patients with UCLP. Such surgeries not only correct structural anomalies but also contribute to significant changes in the upper airway, leading to enlarged airway passages and reduced resistance [10]. However, performing maxillary advancement presents several challenges, including soft tissue scarring, compromised bone quality, and irregular dentition [11]. Surgical planning must consider various critical factors like the extent of advancement needed, control over the palatal plane vector, and the positioning of the upper incisors to optimize outcomes for patients with these conditions [12].

Enhanced nasal airflow post-surgery can have significant implications, potentially enhancing olfaction, sinus drainage, sleep comfort, and physical activity engagement [9,13,14,15,16].

Computational Fluid Dynamics (CFD) serves as a powerful tool to analyze complex fluid flow phenomena, including the study of the nasal airway [17,18,19,20,21] and can help us understand the surgical changes. CFD has particular importance in evaluating patients with nasal obstruction and other nasal symptoms [20,22]. Traditionally, the assessment of the nasal airway includes subjective assessment tools such as the Nasal Obstruction Symptom Evaluation (NOSE) [23]. In many cases, however, patients may never have experienced normal airway flow and subjective assessment may lead to under-reported airway obstruction and discomfort. CFD emerges as a superior tool because it provides objective measurements that can correlate with how patients report their nasal breathing experiences. It is capable of analyzing nasal airflow and measuring additional variables such as heat transfer or nasal wall shear stress. These measurements are particularly useful as they reflect the activity of nasal trigeminal sensitive endings, which are critical in sensing nasal airflow [20,24]. CFD’s potential extends to personalized nasal surgeries, predicting biophysical outcomes that aid surgical planning [22]. CFD allows the simulation of virtual surgeries to predict changes in airflow following hypothetical modifications of the nasal structure. This capability aids surgeons in planning and selecting the most appropriate surgical techniques tailored to the individual characteristics of each patient’s nose.

In particular, the integration of CFD simulations with patient-specific CT scans has exceptional promise in modeling and predicting airflow dynamics, especially in the realm of surgical planning and outcome assessment [20,24,25]. Studies using CFD simulations based on CT scan data have been conducted to analyze nasal airflow [26], predict nasal patency [27], and explore changes in nasal airflow and heat transfer post-surgery for nasal obstructions [28]. By integrating patient-specific CT scans with CFD simulations, researchers can offer a detailed, quantitative, non-invasive, and objective analysis of nasal airflow, circumventing the limitations inherent to subjective measures of patient comfort or other traditional assessment techniques. This approach aligns closely with experimental measures of well-being, offering a critical lens through which to assess the efficacy of surgical interventions in UCLP cases [20,22,24,25,28,29,30,31,32,33].

Previous studies have employed CFD to investigate the effects of septoplasty, turbinate surgery, and endoscopic sinus surgery on nasal airflow and resistance [17,18,19,20,21]. These models have been pivotal in studying the impact of procedures like septoplasty on nasal airflow, demonstrating improvements in airflow distribution and particle deposition post-surgery [34]. CFD models have proven reliable for evaluating subjective nasal patency against objective airflow and mucosal cooling metrics [29]. CFD has also been utilized in the study of UCLP nasal airway [35,36,37,38]. Notably, Frank-Ito et al. used computational modeling to analyze nasal airway obstruction in subjects with unilateral cleft lip nasal deformity (uCLND). They found significant differences in nasal cavity asymmetry, resistance, and airflow partition between the affected and unaffected sides compared to non-cleft subjects with nasal obstruction. The results highlight the severity and specificity of nasal issues in uCLND patients compared to other patients with nasal obstructions [35]. Iwasaki et al.’s retrospective study evaluated the effects of rapid maxillary expansion (RME) on nasal airway ventilation in children with UCLP. The study reported significant improvements in nasal airway parameters like pressure, velocity, resistance, airflow rate, and cross-sectional area, demonstrating RME’s effectiveness in enhancing nasal ventilation in UCLP children [37,38]. Traditional subjective assessments often do not capture the full range of functional changes, particularly in children, where subjective symptom reporting may be challenging. Therefore, the use of CFD for detailed and accurate evaluation of pediatric nasal anatomy is important.

This study aims to utilize CFD to assess the biophysical effects on nasal airway dynamics before and after LeFort I osteotomy advancement in patients with UCLP. By employing state-of-the-art CFD coupled with detailed patient-specific CT imaging data, our goals are to quantitatively assess both the morphological and airflow dynamic changes pre- and post-surgery. Specifically, we aim to measure variables such as nasal airway volume, cross-sectional area, airflow velocity, pressure distribution, volumetric flow rate, and nasal resistance. By capturing these comprehensive metrics, we seek to evaluate the effectiveness of surgical interventions in terms of improved airway patency and reduced airflow resistance. This approach provides a robust framework for assessing the biophysical impacts of LeFort I osteotomy, ultimately contributing to enhanced surgical planning and better postoperative outcomes for patients with UCLP.

## 2. Materials and Methods

### 2.1. Study Design and Participants

A retrospective preliminary study was conducted at the Children’s Medical Center Dallas. Models were developed to assess the difference in nasal airways between a patient with cleft lip and palate, before and after surgery, and an age-matched control with no craniofacial deformities or respiratory disorders. The Institutional Review Board of the University of Texas Southwestern approved all CT scans that were used in the study. All scans were obtained for clinical purposes using a Siemens SOMATOM Definition CT scanner (Erlangen, Germany) following the Craniofacial-Flash protocol detailed in [39]. The Siemens SOMATOM Definition AS CT scanner is a third-generation helical, multislice unit. The voxel size for the CT scans was $0.4102\times 0.4102\times 1.5\;{\mathrm{mm}}^{3}$, with dimensions of 210 mm width, 210 mm height, and 190.5 mm depth. The protocol used a pitch of 3 and tube voltage ranged between 70 and 100 kVp. The patient was a male at the age of 13.09 years when the pre-surgery scan was taken and at the age of 16.85 years when the post-surgery scan was taken. The healthy control was a female at the age of 17.85 years when the scan was taken. The surgical procedure involved LeFort I osteotomy with a maxillary advancement of approximately 6 mm, which is within the typical range for such corrective surgeries.

### 2.2. 3D Reconstruction

The CT scans were processed using MIMICS V25.0 (Materialise Interactive Medical Image Control System), a software suite designed for the segmentation and 3D reconstruction of anatomical structures from medical imaging data. The segmentation process began with the identification of the nasal airway. Thresholding was applied to isolate the airway from the surrounding soft and hard tissues, with threshold values determined based on Hounsfield units specific to air and soft tissue contrast. Following thresholding, region growing and manual editing were used to refine the segmentation, ensuring accurate delineation of the nasal airway boundaries. After segmentation, the software generated a 3D reconstruction of the nasal airway by converting the segmented slices into a continuous 3D model. Any discontinuities or artifacts in the segmentation were addressed using manual correction tools available in MIMICS. The final 3D models of the nasal airway were then exported as STL (stereolithography) files, which were subsequently used for further analysis.

### 2.3. Nasal Airway Geometry Modeling

The STL files were imported into SolidWorks 2023 (Dassault Systemes, Vélizy-Villacoublay, France), a computer-aided design (CAD) software package to create a suitable volume for simulation. This involved generating cross-sectional sketches based on the profile of the nasal airway scans. Subsequently, solid volumes were generated using cross-sectional sketches of the nasal airway. Several assumptions were made during the geometry creation process, such as treating the nasal mucosa as a rigid surface and neglecting the presence of nasal hairs and mucus. These assumptions were made to simplify the modeling process and reduce computational complexity.

### 2.4. Computational Fluid Dynamics Simulations

Computational fluid dynamics (CFD) simulations were carried out using Ansys Fluent 2022, a leading commercial CFD software package. The simulations were aimed at analyzing the quasi-steady-state airflow through nasal airway geometries under laminar flow conditions. The governing equations for the flow, the continuity, and the Navier–Stokes equations are given by:

(1) $$\nabla \cdot \vec{U}=0$$

(2) $$\rho \left(\frac{\partial \vec{U}}{\partial t}+\left(\vec{U}\cdot \nabla \right)\vec{U}\right)=-\nabla P+\mu \nabla^{2}\vec{U}$$

where $\vec{U}$ is the velocity vector of the fluid domain, *P* denotes pressure, ρ is the fluid density, and μ represents the dynamic viscosity. Nasal airway geometries were first designed in SolidWorks and then imported into Ansys Fluent 2022 and converted into volume meshes using tetrahedral elements. The average element size was set to 0.5 mm, and mesh quality metrics, including aspect ratio, skewness, and orthogonality, were carefully monitored to ensure the reliability of the mesh. Boundary conditions were defined with a constant pressure inlet at the nostrils and a constant pressure outlet at the nasopharynx to mimic typical physiological conditions during quiet breathing. The nasal mucosa was modeled with a no-slip wall boundary condition. The simulations assumed negligible mucous effects due to their minimal thickness and low velocity. Air, considered as the working fluid, was modeled under standard atmospheric conditions with a density (ρ) of 1.204 kg/m^3^ and a dynamic viscosity (μ) of $1.825\times 10^{-5}$ kg/ms.

In addition to laminar flow simulations, turbulent flow conditions were modeled using the realizable k−ϵ turbulence model with standard wall functions. This model was chosen for its ability to more accurately predict the flow characteristics in regions where adverse pressure gradients and separation might occur, which are common in the complex geometries of postoperative nasal airways.

The realizable k−ϵ model introduces refined transport equations for turbulent kinetic energy (*k*) and its dissipation rate (ϵ), enhancing the simulation’s capacity to capture the effects of turbulence within the nasal cavity. The model equations are as follows:

(3) $$\frac{\partial (\rho k)}{\partial t}+\nabla \cdot \left(\rho \vec{U}k\right)=\nabla \cdot \left(\frac{\mu_{t}}{\sigma_{k}}\nabla k\right)+P_{k}-\rho \epsilon$$

(4) $$\frac{\partial (\rho \epsilon )}{\partial t}+\nabla \cdot \left(\rho \vec{U}\epsilon \right)=\nabla \cdot \left(\frac{\mu_{t}}{\sigma_{\epsilon }}\nabla \epsilon \right)+C_{1\epsilon }\frac{\epsilon }{k}P_{k}-C_{2\epsilon }\rho \frac{\epsilon^{2}}{k}$$

where $P_{k}$ represents the production of turbulent kinetic energy, $\mu_{t}$ is the turbulence viscosity, and $\sigma_{k}$, $\sigma_{\epsilon }$, $C_{1\epsilon }$, and $C_{2\epsilon }$ are model constants.

A pressure-based solver was utilized for both laminar steady-state and turbulent analysis with the SIMPLE algorithm for pressure–velocity coupling. The convergence criteria were set to $1\times 10^{-6}$ for all governing equations.

Comparative analyses between preoperative and postoperative anatomies in conjunction with an age-matched control during steady inspiration were performed. The comparisons were standardized either by the volumetric flow rate (*Q*) or by the pressure difference (Δp) between the inlet and outlet. Nasal resistance was calculated using the formula Δp/Q (Pa/s/mL), where Δp signifies the pressure drop from the nostrils to the choana and *Q* denotes the volumetric flow rate.

## 3. Results

This investigation quantitatively evaluated the efficacy of surgical interventions on nasal airway resistance among UCLP patients, focusing on variations between the cleft (left) and non-cleft (right) sides pre- and post-surgery, alongside comparisons with control values.

### 3.1. Geometry and Structural Changes

**Figure 1:**
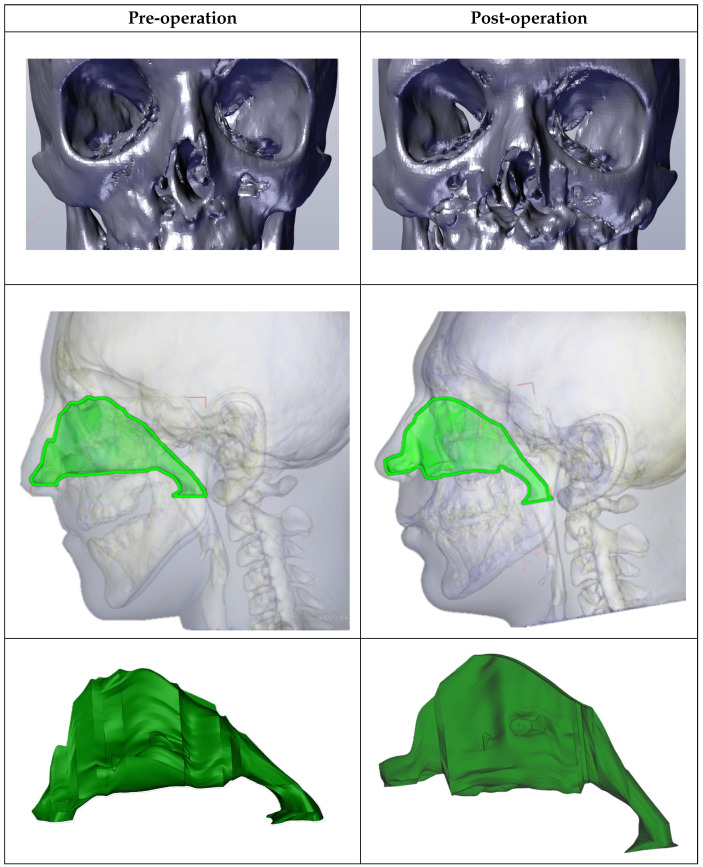
Comparison between the nasal cavity pre- and post-op.

**Figure 2:**
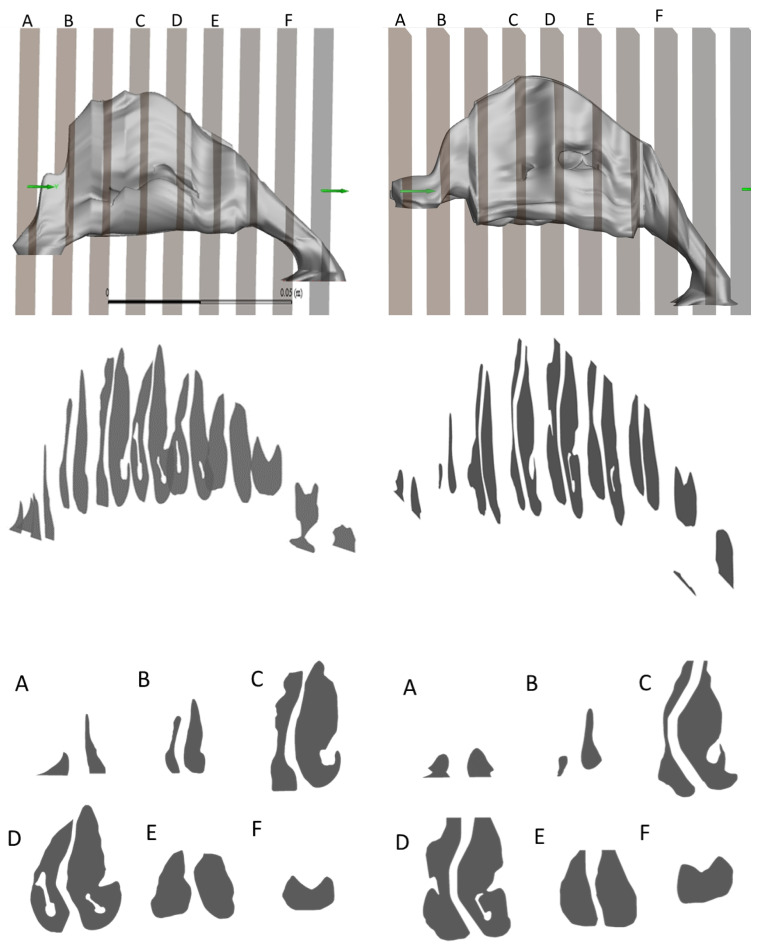
Comparison of the cross-section view of nasal cavity pre- and post-op.

Alterations in nasal cavity geometry were identified post-surgery. The cross-sections were taken at anatomically consistent landmarks such as the anterior nasal spine, the posterior end of the hard palate, and the nasopharynx to ensure comparability between pre- and post-surgery models. The cross-sectional analysis provided in Figure 1 and Figure 2 highlights these changes.

Cross-sectional surfaces were obtained from sections in Ansys Fluent. Planes perpendicular to the inlet at the outlet plane were placed at fixed intervals along the Nasal Airway in Ansys Fluent and sections were obtained. The preoperative model of the UCLP nasal deformity showed a significantly altered geometry compared to that of the control. On average, the cross-sectional area of the preoperative model was 34.11% smaller than that of the control, with percentage differences in each plane ranging from 18.1% to 61.5%. This variation underscores the pronounced structural changes in the patient’s nasal anatomy. Both the preoperative model and the postoperative model demonstrated distinct trends in their cross-sectional areas.

The volume of the postoperative model was calculated to be 35.2 cm^3^, representing a significant increase from the preoperative volume of 27.8 cm^3^. In comparison, the volume of the nasal airway in the control was 47.4 cm^3^. The increased cross-sectional area is particularly significant because it directly correlates with decreased airway resistance and enhanced airflow. This geometric expansion helps to normalize the airflow patterns to more closely resemble those observed in non-cleft subjects. Trends observed in the postoperative models indicated a progressive normalization towards the control models, suggesting effective surgical outcomes.

### 3.2. Turbulent Kinetic Energy (TKE) and Shear Stress Analysis

Analysing Turbulent Kinetic Energy (TKE) and shear stress provides vital insights into the dynamics of nasal airflow and the mechanical forces that influence mucosal health and airflow comfort post-surgery. Turbulence within the nasal airflow significantly affects the overall resistance and sensation of breathing, with implications for both physiological function and patient comfort.

Our findings demonstrate a noteworthy reduction in TKE following surgical intervention. Specifically, the mean percentage reduction in TKE from preoperative to postoperative conditions was −20.93%. This decrease suggests a smoother airflow pattern, which can contribute to a more comfortable breathing experience for patients. Comparing preoperative conditions to the control (non-cleft subject), TKE was reduced by a mean percentage difference of −39.05%. These changes are illustrated in Table 1, showing the convergence of postoperative TKE levels towards those observed in the control, indicating a successful surgical outcome in terms of normalizing airflow dynamics. The reduction in TKE post-surgery is indicative of diminished airflow turbulence, which is generally associated with smoother and less obstructive air passage through the nasal cavity.

### 3.3. Turbulence Intensity Analysis in Nasal Airway Flow

The analysis of turbulence intensity in nasal airflow is crucial for understanding the mechanical environment within the nasal cavity, both before and after surgical intervention. Turbulence intensity, quantified as the variation in airflow velocity, directly impacts the shear stress exerted on the nasal mucosa and is a key factor in evaluating the aerodynamic efficiency of nasal passages.

**Figure 3:**
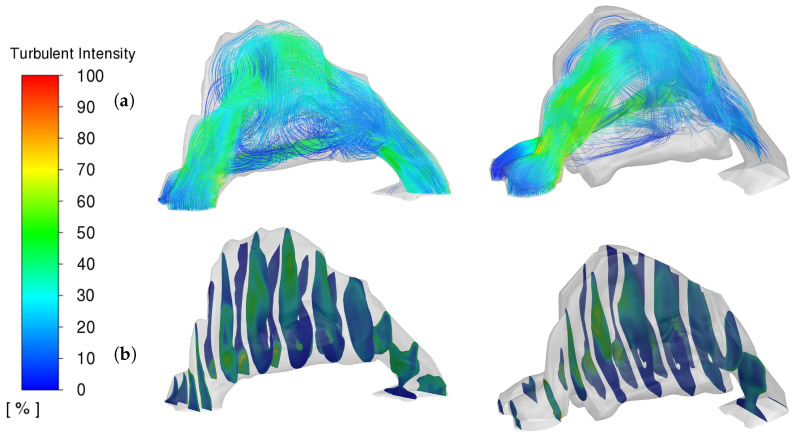
Comparative Analysis of Nasal Airway Turbulence Intensity (**a**) Streamlines, (**b**) Cross-Sectional Contours. Pre-op (**LEFT**) Post-op (**RIGHT**).

In our study, we observed that both preoperative and postoperative models exhibited similar peak turbulence intensities, with maximum values reaching up to 65%. However, notable differences were evident in the spatial distribution of these intensities within the nasal cavity. Figure 3 illustrates these variations, where panel (a) shows streamline patterns and panel (b) details the cross-sectional contours of turbulence intensity.

The preoperative model displayed increased regions of high turbulence intensity, particularly towards the posterior region of the nasal airway leading into the nasopharynx. This suggests a higher degree of airflow disruption in the preoperative state, potentially contributing to increased nasal resistance and reduced patient comfort. Conversely, the postoperative model, while maintaining similar peak values, showed a more uniform distribution of lower-intensity values. This indicates a smoother airflow pattern post-surgery, likely resulting from surgical modifications that enhance the aerodynamic properties of the nasal cavity.

These observations are critical as they suggest that surgical interventions, while not significantly altering the peak turbulence intensity, effectively modify the airflow dynamics to reduce areas of high turbulence, especially in critical regions affecting breathing comfort and efficiency. Such insights are invaluable for designing surgical strategies aimed at optimizing nasal airflow and ensuring mucosal health.

This comprehensive analysis underscores the importance of considering not just the peak values of turbulence intensity but also their distribution within the nasal cavity to fully assess the impact of surgical interventions on nasal airflow dynamics.

### 3.4. Wall Shear Stress Distribution Analysis

The evaluation of wall shear stress in the nasal cavity provides significant insight into the mechanical forces exerted by airflow, which are critical for understanding mucosal irritation and overall nasal health. Wall shear stress, measured in Pascals (Pa), reflects the frictional force caused by airflow interacting with the nasal walls and has direct implications for the comfort and health of nasal tissues.

Shear stress, particularly on the nasal mucosa, is crucial as it influences tissue integrity, susceptibility to injury, and the sensation of airflow. The average preoperative shear stress was measured to be 0.085 N/m^2^, reducing to 0.0742 N/m^2^ postoperatively, and further to 0.0637 N/m^2^ in the control model. This reduction is beneficial as lower shear stress on the nasal mucosa reduces the risk of mechanical irritation and potential damage, thereby enhancing patient comfort and mucosal health. Reduced shear stress postoperatively suggests that the nasal mucosa is subjected to less mechanical force, which may decrease the risk of inflammation and other complications associated with high shear stress, such as dryness and crusting.

**Figure 4:**
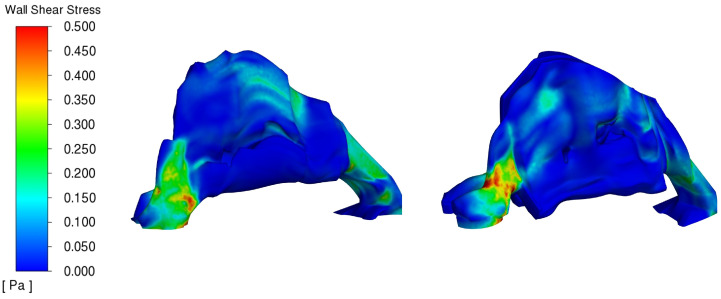
Comparative Analysis of Nasal Airway Wall Shear Stress Distribution Pre-op (**LEFT**) Post-op (**RIGHT**).

Our contour map analysis, as shown in Figure 4, details the distribution of wall shear stress within the nasal airway, ranging from 0 to 0.5 Pa in both the preoperative and postoperative conditions. This visualization allows a detailed comparison of the mechanical environment before and after surgical intervention.

The data reveal that both the preoperative and postoperative models exhibit similar peak shear stress values, with high stress levels concentrated in the nostrils and the anterior part of the nasal cavity, reaching up to 0.5 Pa. This area experiences the greatest airflow impact due to its direct exposure to incoming air, making it a critical region for assessing surgical outcomes. Further into the nasal cavity, particularly towards the posterior heading into the nasopharynx, shear stress levels are approximately 0.3 Pa.

Interestingly, the preoperative model shows a slightly wider distribution of high shear stress areas compared to the postoperative model. This indicates more extensive regions of elevated mechanical stress on the nasal mucosa pre-surgery, which can contribute to discomfort and potential mucosal damage. The postoperative improvements suggest a strategic reduction in these high-stress regions, likely achieved through surgical modifications designed to streamline airflow and reduce frictional forces against the nasal walls.

This contour map analysis underscores the importance of surgical intervention in not only maintaining peak shear stress levels but also more importantly, in modifying the distribution of these stresses to enhance patient comfort and nasal function. The reduction in the extent of high shear stress areas post-surgery demonstrates an effective optimization of nasal airflow dynamics, contributing to better overall nasal health and patient outcomes.

Our analysis also encompassed both laminar and turbulent models of nasal resistance. These models are essential for understanding the ease with which air flows through the nasal passages. Nasal resistance analyses demonstrated strong agreement between laminar and turbulent models. Average values were calculated over −5 to −45 Pa. Both models indicated an overall decrease in nasal airway resistance, facilitating easier breathing and potentially reducing the energetic cost of respiration for the patient.

### 3.5. Velocity Dynamics

The study of airflow velocity within the nasal cavity provides critical insights into the functional outcomes of surgical interventions in UCLP patients. The velocity profiles, particularly how they change pre- and post-surgery, are indicative of the surgical impact on nasal airway patency and resistance.

**Figure 5:**
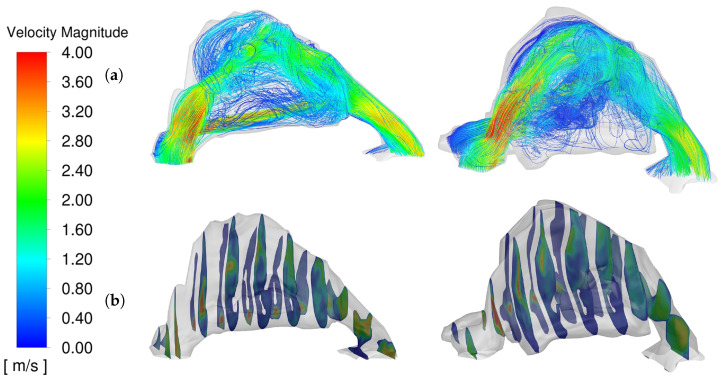
Comparative Analysis of Nasal Airway Velocity Profiles (**a**) Velocity Streamlines, (**b**) Cross-Sectional Velocity Magnitude Contours. Pre-op (**LEFT**) Post-op (**RIGHT**).

Prior to surgical intervention, the velocity distribution within the nasal cavity of the UCLP patient displayed significant variations. The average airflow velocity in the preoperative models was recorded at 2.8 m/s. Peak velocities were particularly high, reaching up to 4.1 m/s, primarily observed in the narrower sections of the affected side’s nasal cavity. These high velocities are symptomatic of increased airway resistance, typically due to structural constriction and irregularities inherent in cleft conditions. The streamlines depicted in Figure 5a illustrate these high-speed flows entering through the nostrils and then decelerating as they encounter the broader nasal cavity, only to accelerate again in the constricted regions towards the nasopharynx. The velocity magnitude contours in Figure 5b further underscore the uneven distribution of airflow, highlighting areas of potential turbulence and increased shear stress on the nasal mucosa.

The postoperative model reveals a stark contrast in the airflow dynamics within the nasal cavity. Following surgical corrections, the average velocity decreased to 2.3 m/s, suggesting a broadening of the airway and a reduction in structural impediments. Despite the reduction in average velocity, peak velocities remained comparable to preoperative measurements, indicating that while overall resistance may be reduced, localized areas of higher velocity still exist. These may be due to residual structural irregularities not completely corrected by surgery or normal anatomical variations in airways.

The streamlines in the postoperative model demonstrate a more streamlined flow, with less pronounced velocity gradients than in the preoperative condition. This streamlined flow suggests an improvement in airway patency, reducing the energy expenditure required for breathing and potentially enhancing patient comfort. The velocity magnitude contours show a more uniform distribution across different sections of the nasal cavity, indicating a more homogeneous flow pattern that aligns more closely with normal physiological conditions.

The analysis of velocity profiles pre- and post-surgery elucidates the aerodynamic improvements achieved through surgical interventions. The shift from high, localized velocities and pronounced gradients to a more even velocity distribution signifies a decrease in overall nasal airway resistance. This decrease contributes to more effective and comfortable breathing patterns for patients. Moreover, the consistency in postoperative velocity magnitudes across the nasal cavity suggests a successful surgical expansion of critical narrow points, which likely contributes to the reduced symptoms of nasal obstruction and improved overall nasal function.

### 3.6. Pressure Dynamics

The static pressure within the nasal airway is a crucial metric for assessing the aerodynamic performance and overall health of the respiratory pathway, particularly in UCLP patients who undergo surgical correction. By analyzing the pressure dynamics, we can better understand the extent of surgical improvements in airway resistance and patient comfort.

**Figure 6:**
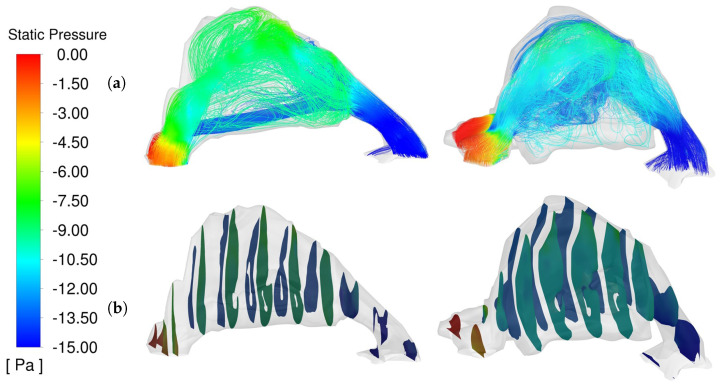
Comparative Analysis of Nasal Airway Static Pressure (**a**) Static Pressure Streamline, (**b**) Cross−Sectional Pressure Contours. Pre−op (**LEFT**) Post−op (**RIGHT**).

In the preoperative condition, the nasal cavity of the affected side exhibited significantly elevated static pressure zones, which were about 200% higher than those on the unaffected side, as depicted in Figure 6. This imbalance highlights the constriction and irregular geometry typically associated with UCLP, which impede the airflow, thereby increasing the static pressure within the narrow passages. At the nostril or inlet, the pressure was approximately 0 Pa, corresponding to atmospheric pressure. As the air moved deeper into the nasal cavity, it encountered increased resistance due to structural narrowings, leading to a notable pressure drop, which reached −15 Pa at the outlet. Following surgical intervention, the pressure dynamics within the nasal cavity showed significant improvement. The postoperative models demonstrated a more balanced and reduced pressure gradient across the nasal cavity. The affected side still showed higher pressures compared to the unaffected side, but the difference was considerably less pronounced than in the preoperative models. This reduction in pressure gradient indicates a successful surgical outcome in which the airway passages were widened or corrected, leading to a more uniform airflow distribution. The cross-sectional pressure contours in Figure 6b validate this observation, showcasing a more consistent pressure profile that more closely resembles physiological norms.

### 3.7. Volumetric Flow Rate and Nasal Resistance

Volumetric flow rate and nasal resistance are both critical factors in assessing nasal obstructions and the functional efficacy of surgical interventions in patients.

**Figure 7:**
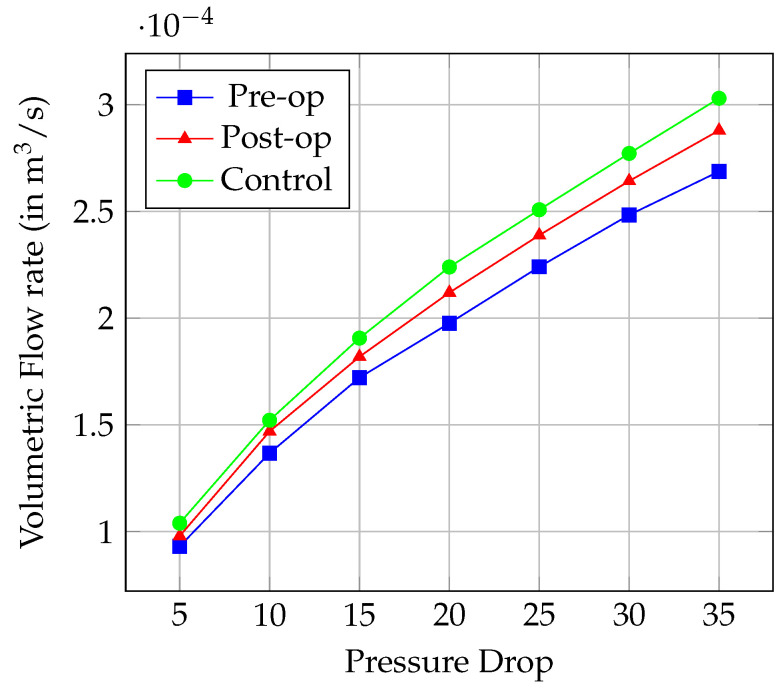
Comparison of Volumetric Flow rate vs. Pressure Drop.

The data presented in Figure 7 show that postoperative volumetric flow rates have improved across all levels of pressure drop compared to preoperative values. Specifically, the average increase in volumetric flow rate post-surgery was 6.53%. This indicates a significant enhancement in airway patency, allowing a greater volume of air to pass through the nasal cavity at lower pressure gradients. Although the control group demonstrated an average flow rate increase of 11.91%, the postoperative improvements approach these control values, reflecting a near-normalization of nasal airflow dynamics. This trend suggests that while the surgical outcomes significantly improve nasal airflow, there remains a slight gap when compared to the ideal, non-cleft-affected nasal airway.

The changes in nasal resistance further corroborate the improvements in airflow dynamics observed in the flow rate analysis. As shown in Table 2, there is a consistent decrease in nasal resistance across all measured pressure drops postoperatively, with an average reduction of 6.23% from preoperative values. This decrease highlights the surgical success in reducing anatomical obstructions and enhancing the overall efficiency of the nasal airway. The control group still maintains the lowest nasal resistance, indicative of an optimal airway structure without congenital deformities.

## 4. Discussion

Understanding the intricate relationship between pressure, airflow, and nasal resistance can be beneficial to understanding how nasal physiology relates to breathing difficulties, particularly in patients with unilateral cleft nasal deformities. Our study observed improvements in cross-sectional area and volume in the nasal cavity following LeFort I osteotomy advancement, which consequently modulated airflow speed and reduced nasal resistance under physiological conditions.

The asymmetry of the nasal cross-sectional area in patients with UCLP, as reported by Frank-Ito et al., is attributed to middle-to-posterior septal deviation [35]. Marcus et al. also identified multiple sites of potential obstruction in patients with UCLP that lead to increased resistance in the anterior, middle, and posterior regions on both the cleft and non-cleft sides [40]. Frank-Ito et al.’s study also illustrated significant differences in cross-sectional areas of the nasal cavities in uCLND subjects compared to normal subjection and subjects with nasal obstruction. Their use of CFD revealed notable differences in flow partition and resistance, suggesting pronounced airflow dysfunction that contributes to the challenges in achieving nasal patency. Frank-Ito et al. also utilized subjective NOSE scores to assess symptom severity and were able to correlate them to CFD-computed nasal resistances [35]. Similarly, Iwasaki et al. found increased nasal resistance and maximal upper airway pressure in UCLP patients compared to controls [38]. Our findings are consistent with these results, showing that preoperatively, patients had greater resistance and decreased flow rate compared to control subjects. Ramanathan et al. also reported significant decreases in nasal resistance, wall shear stress, and pressure following septoplasty, as assessed using CFD [41].

The reduction in nasal resistance is closely associated with the post-surgery increase in the cross-sectional area and volume of the nasal cavity. A larger cross-sectional area enables smoother airflow, reducing pressure drops and localized pressure zones, thereby enhancing the patient’s breathing ability, comfort, and overall quality of life. Iwasaki et al. have reported the impact of maxillary alterations in patients with UCLP, demonstrating that rapid maxillary expansion results in an increase in both the cross-sectional area and the airflow rate [37]. Wang et al. conducted acoustic rhinometry in patients with UCLP undergoing combined LeFort I and septoplasty, revealing enhancements in minimal cross-sectional area, nasal resistance, and nasal volumes [42]. In a related study, Yatabe-Ioshida et al. observed an increase of 20% in nasopharynx volume post-LeFort I osteotomy in patients with UCLP [43]. Furthermore, Zhai et al. reported a 13.85% augmentation in nasal cavity volume [44]. Corroborating these findings, our postoperative data indicate a mean increase of 26.6% in nasal volume, accompanied by a 6.53% elevation in airflow rate and a 6.23% reduction in resistance subsequent to the surgical intervention. Ghoneima et al. [45] did both laminar and turbulent simulations of the upper airway after rapid maxillary expansion and measured increases in nasal cavity volume and nasopharynx volume. They also found decreases in pressure, velocity, and turbulent kinetic energy in both laminar and turbulent flow. They found a similar agreement between laminar and turbulent results for flow variables. Zhu et al. [46] also did a turbulent simulation of patients with septal deviation and reported increased shear stress and nasal resistance relative to their healthy control. These findings also validate the agreement we found between laminar and turbulent simulations under quiet breathing conditions.

In contemporary clinical practice, the evaluation of nasal airway obstruction (NAO) associated with cleft deformities typically relies on subjective assessment tools. These include the Nasal Obstruction Symptom Evaluation (NOSE) [23] questionnaires, the Facial Aesthetic Patient-Reported Outcome (FACE-Q) instrument [47], and the Cleft Evaluation Profile [48], alongside traditional physical examinations. While these methods are valuable for capturing patient and caregiver perceptions and leveraging the experiential knowledge of surgeons, they inherently lack the ability to objectively quantify physiological parameters such as nasal resistance, shear stress, or nasal airflow. Techniques like acoustic rhinometry provide objective measurements of nasal geometry and airflow resistance [49,50,51]. However, acoustic rhinometry is unable to directly measure airflow, which limits its ability to provide a comprehensive assessment of nasal function [49,50,51].

This gap underscores the significant potential of Computational Fluid Dynamics (CFD) modeling in the realm of nasal patency evaluation. Unlike subjective assessments, CFD provides a detailed, quantitative analysis of airflow dynamics and resistance within the nasal cavity and unlike acoustic rhinometry, it can give us a holistic picture of nasal obstruction. By simulating various physiological conditions and surgical modifications, CFD can offer insights that are not readily obtainable through conventional clinical evaluations.

While this study provides valuable insights into the benefits of LeFort I advancement for patients with UCLP, several limitations must be acknowledged. A primary constraint is the limited sample size, as patients at our institution do not routinely receive pre- and postoperative standard CT scans [39]. Future studies with larger sample sizes and more diverse patient populations are essential for further validation of our findings. The voxel size of 1.5 mm used in the CT scans was selected to balance the need for high-resolution imaging and minimizing radiation exposure, especially in the pediatric population. While this resolution is generally adequate for clinical purposes, finer anatomical details might be missed, which could potentially affect the precision of the CFD predictions. Future studies could benefit from higher-resolution imaging, albeit with careful consideration of radiation dose, to enhance the geometric accuracy of the models. Additionally, the continuous growth and development of pediatric patients may mean that current anatomical structures derived from CT scans might not reflect long-term outcomes due to the evolving nature of the pediatric airway. Moreover, CFD models do not encompass the full complexity of the human nasal airway. Factors such as mucosal inflammation and nasal secretions can affect airflow dynamics and resistance [17,18,19]. The rigidity of the nasal cavity walls was also assumed in our model. This approximation generally holds for the bony structures of the nasal cavity; however, movement of soft tissue could contribute effects not currently accounted for by our CFD approach. The study also relied on quasi-steady-state laminar and k−ϵ turbulent CFD simulations, which may not fully represent transient effects or turbulence observed during actual breathing [20]. Additionally, the smoothing of the nasal cavity model to reduce digitalization artifacts introduces limitations that may have led to underestimations of nasal resistance in our simulations, while this process eliminates unrealistic patterns created by digitalization, it also results in a much smoother surface with reduced friction compared to the actual nasal cavity. In reality, the walls of the nasal cavity are covered by mucosa and nasal hair in the anterior nose, contributing to a more irregular surface and potentially higher friction [19].

## 5. Conclusions

This study effectively underscores the significant challenges encountered by individuals afflicted with unilateral cleft lip and palate (UCLP) and elucidates the transformative impacts of LeFort I osteotomy on their nasal airflow dynamics. Through comprehensive Computational Fluid Dynamics (CFD) analyses, substantial post-surgical enhancements in nasal cavity geometry, airflow velocity, pressure dynamics, volumetric flow rate, and nasal resistance were demonstrated. The surgery resulted in an expanded nasal cavity volume, increased cross-sectional area, and decreased nasal resistance, indicating enhanced breathing efficiency and improved patient comfort. Limitations of this study include the resolution of CT scans, small sample size, and lack of experimental validation. These findings provide a quantitative framework for evaluating the efficacy of surgical interventions, aiding in both preoperative planning and postoperative assessments.

## Figures and Tables

**Table 1:** Summary of Surgical Intervention Effects on Nasal Airflow Dynamics.

Parameter	Pre-op	Post-op	Control
Laminar Shear Stress (N/m^2^)	0.085	0.074	0.064
Turbulent Shear Stress (N/m^2^)	0.088	0.075	0.064
Laminar Resistance (Pa·s·cm^3^)	0.105	0.102	0.090
Turbulent Resistance (Pa·s·cm^3^)	0.104	0.099	0.089
TKE (m^2^/s^2^)	0.180	0.142	0.109

**Table 2:** Nasal Resistance (Pa·s·mL^−1^) Across Different Models.

Pressure Drop (Pa)	Pre-op	Post-op	Control
5	0.0537	0.0511	0.0481
10	0.0731	0.068	0.0657
15	0.0871	0.0825	0.0787
20	0.101	0.0944	0.0893
25	0.112	0.105	0.0997
30	0.121	0.114	0.1082
35	0.130	0.122	0.1154
Average	0.0968	0.091	0.0864

## Data Availability

The datasets presented in this article are not readily available because they contain Protected Health Information (PHI) which is subject to strict privacy regulations. Access can only be granted under specific conditions that ensure compliance with privacy laws and ethical guidelines.
